# Simultaneous determination of cetirizine, phenyl propanolamine and nimesulide using third derivative spectrophotometry and high performance liquid chromatography in pharmaceutical preparations

**DOI:** 10.1186/s13065-017-0326-9

**Published:** 2017-10-05

**Authors:** Fatma Ahmed Aly, Nahed EL-Enany, Heba Elmansi, Amany Nabil

**Affiliations:** Department of Analytical Chemistry, Faculty of Pharmacy, University of Mansoura, Mansoura, 35516 Egypt

**Keywords:** Third derivative spectrophotometry, HPLC, Cetirizine (CTZ), Phenylpropanolamine (PPA), Nimesulide (NMS), Tablets

## Abstract

**Background:**

The combination between cetirizine (CET), phenylpropanolamine (PPA) and nimesulide (NMS) under trade name Nemeriv Cp tablet is prescribed for nasal congestion, cold, sneezing, and allergy. Among all published methods for the three drugs; there is no reported method concerning estimation of CTZ, PPA and NMS simultaneously and this motivates us to develop new and simple methods for their assay in pure form and tablet preparations.

**Results:**

Two new methodologies were described for the simultaneous quantification of cetirizine (CTZ), PPA and NMS. Spectrophotometric procedures relies on measuring the amplitudes of the third derivative curves at 238 nm for CTZ, 218 nm for PPA and 305 nm for NMS. The calibration graphs were rectilinear over the ranges of 8–90 µg/mL for CTZ, 20–100 µg/mL for PPA and 20–200 µg/mL for NMS respectively. Regarding the HPLC method; monolithic column (100 mm × 4.6 mm i.d) was used for the separation. The used mobile phase composed of 0.1 M phosphate buffer and methanol in the ratio of 40:60, v/v at pH 7.0. The analysis was performed using UV detector at 215 nm. Calibration curves showed the linearity over concentration ranges of 5–40, 10–100 and 10–120 µg/mL for CTZ, PPA and NMS.

**Conclusion:**

Application of the proposed methods to the laboratory prepared tablets was carried out successfully. The results were compared with those obtained from previously published methods and they were satisfactory.

**Graphical abstract:**
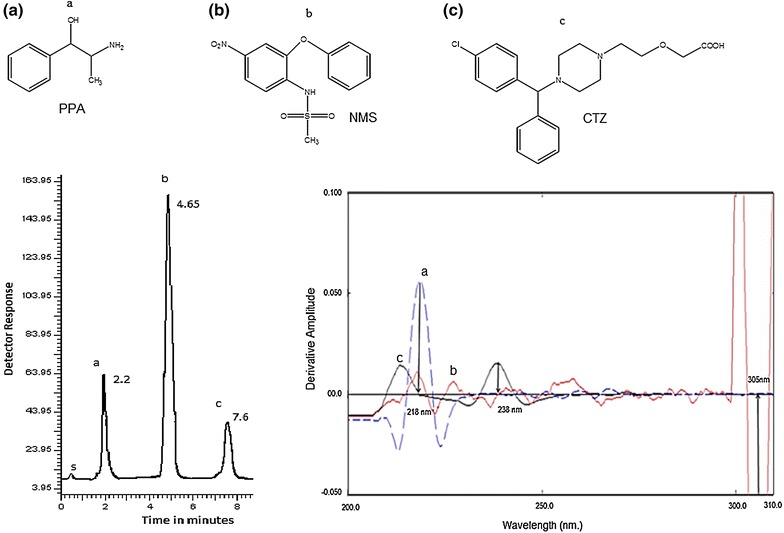
Graphical abstract represents the chemical structures, representative chromatogram for the HPLC separation of **a** PPA, **b** NMS and **c** CTZ and third derivative absorption spectra of **a** PPA, **b** NMS and **c** CTZ for the spectrophotometric method.

## Introduction

**Fig. 1:**
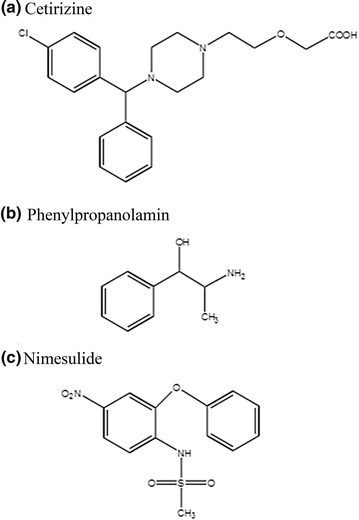
The structural formulae of the studied drugs. **a** Cetirizine, **b** phenylpropanolamine, **c** nimesulide

Cetirizine (CTZ, Fig. 1a); is non-sedating antihistamine with long acting activity for treatment of urticarial and rhinitis [1]. It is ([2-[4-[(4-chlorophenyl) phenylmethyl]-1-piperazinyl] ethoxy] acetic acid). The BP suggested a potentiometric titration method for determination of CTZ in its pure form; while it recommended an HPLC method for both cetirizine oral solution and tablets [2]. Different analytical procedures were reported for its determination including HPLC [3–6], HPTLC [7], capillary electrophoresis [8] and spectrophotometry [9].

Phenylpropanolamine hydrochloride (PPA, Fig. 1b) is a nasal decongestant mainly used in combinations for relief of cold symptoms as it has indirect sympathomimetic activity [1]. Its chemical name is (1RS, 2SR)-2-amino-1-phenylpropan-1-ol. The BP described non aqueous potentiometric titration for PPA [2]. The USP suggested non-aqueous titration method using glacial acetic acid for PPA pure form and HPLC method for its capsules, extended released capsules, tablets, extended released tablets and oral solutions [10]. There are different methods used for PPA determination as HPLC [5, 6, 11], capillary gas chromatography [12], spectrophotometry [13] and flow injection [14] methods.

Nimesulide (NMS, Fig. 1c) is a non-steroidal anti-inflammatory that acts by inhibition of COX-2 enzyme [1]. It is 4′-nitro-2′-phenoxymethanesulphonanilide. The BP mentioned potentiometric titration method for NMS [2]. The literature revealed several methods for NMS determination as HPLC [15–17], spectrophotometry [18] and TLC [19] methods.

The pharmaceutical preparation that contains the three drugs in a tablet dosage form is consisting of (5 mg CTZ, 25 mg PPA and 100 mg NMS) [20]. The current study deals with two simple and sensitive methods for the simultaneous estimation of the three analytes included in this tablet preparation. The spectrophotometric method is a simple and sensitive cost-effective method. It doesn’t need any reagents or other tedious procedures. Although the literature contains two methods for the simultaneous determination of both CTZ and PPA [5, 6]; our proposed HPLC method is superior to the both mentioned methods. Despite Sunil et al. [5] provides an HPLC method for application in plasma and urine, it is less sensitive than our proposed method. Suryan et al. method [6] seeks from the disadvantage of longer retention times, and broader peaks. Our proposed HPLC method, consequently is more sensitive, rapid with sharper peaks than the other mentioned methods owing to the use of monolithic column through this study.

## Experimental

### Apparatus

A Shimadzu (Kyoto, Japan) UV-1601 PC, UV–visible double-beam spectrophotometer was used. The third derivative spectra of the drugs were derived in the wavelength range (200–400) nm using Δλ = 8 nm and scaling factor = 10.

A Shimadzu LC-20 AD prominence liquid chromatograph (Japan) was used for HPLC analysis; with a Rheodyne injector valve and a SPD-20A UV detector set at wave length 215 nm.

### Materials and reagents

Cetirizine hydrochloride pure sample was obtained from Apex Co., Cairo, Egypt (Batch No # 3003CZ8RJ) with 99.95% purity. Phenylpropanolmine hydrochloride (99.88% purity) was kindly brought from Cid Co., Egypt with Batch No # 41204. Nimesulide base was used with purity 99.90% as mentioned by the manufacturer, Batch No # 0006044. It is provided from Pharaonia Co., Alex, Egypt.

Organic solvents (HPLC grade) were purchased from Sigma-Aldrich (Germany).

Sodium hydroxide and sodium dihydrogen phosphate were purchased from ADWIC Co. (Egypt). Orthophosphoric acid (85%, w/v) was provided from Riedel-deHäen (Germany).

### Chromatographic conditions

Chromolith^®^ performance (RP-18 monolithic, 100 mm × 4.6 mm i.d.) is the column used for the investigation. The mobile phase used is a mixture of methanol and buffer (0.1 M phosphate buffer) in a ratio of (60:40 v/v) respectively. The pH was adjusted to be 7. The flow rate was 1 mL/min and the wavelength was 215 nm.

### Standard solutions

CTZ, PPA and NMS 400 µg/mL stock solutions were prepared by dissolving 40 mg of each the studied drugs in 100 mL methanol and further dilution was carried out to achieve the required concentrations for each of the two methods.

### General procedures

#### Construction of calibration graph

##### Spectrophotometric method

Serial dilutions of stock solutions were prepared to give concentrations of 8–90, 20–100 and 20–200 µg/mL for CET, PPA and NMS respectively. The third order derivative amplitudes were measured at 238, 218 and 305 nm for CTZ, PPA and NMS. A plot of the third derivative amplitude against the concentration was constructed to give the calibration curves.

##### Chromatographic method

CTZ, PPA and NMS working standard solutions were prepared by serial dilution of the stock solution in a 10 mL flask to obtain final concentration ranges; 5–40 µg/mL for CTZ, 10–100 µg/mL for PPA, and 10–120 µg/mL for NMS. The solutions were completed to the required volume by the mobile phase and were subjected to the chromatographic analysis under optimum conditions. Calibration graphs were constructed by plotting area under the curve against drug concentration in μg/mL [6–8].

#### Analysis of CTZ, PPA and NMS laboratory-prepared mixtures

Mixtures of CTZ, PPA and NMS in the ratio of 1:5:20 were prepared within the concentration ranges and analysed by the spectrophotometric strategy or the chromatographic strategy under the optimum conditions described in “Chromatographic conditions”. The percent recoveries were determined using regression equations or calibration graphs.

#### Analysis of CTZ, PPA and NMS in their co-formulated tablet

Laboratory co-formulated tablets were prepared as follows; accurately weighed 5 mg CTZ, 25 mg PPA and 100 mg NMS are mixed with 15 mg lactose, 10 mg magnesium stearate, 15 mg maize starch and 20 mg talc. One tablet was weighed, transferred to 100 mL volumetric flask, and completed to the mark with methanol. The solution undergoes 30 min sonication and then filtration till clear solution was obtained clear solution. Aliquots were taken within the concentration ranges for each drug (Table 1), and the chromatographic or spectrophotometric procedure was followed for calculating the percent recoveries [18].

**Table 1:** Analytical performance data for the determination of the studied drugs by the proposed methods

Parameter	3rd Derivative method			HPLC method		
	CTZ	PPA	NMS	CTZ	PPA	NMS
Linearity range (µg/mL)	8–90	20–100	20–200	5–40	10–100	10–120
Intercept (a)	0.006	−0.028	−0.036	1.3 × 10^4^	4.926 × 10^5^	−7.217 × 10^4^
Slope (b)	0.001	0.002	0.002	4.2399 × 10^4^	3.1 × 10^4^	9.343 × 10^4^
Correlation coefficient (r)	0.9999	0.9999	0.9999	0.9999	0.9998	0.9999
S.D. of residuals (S_y/x_)	5.061 × 10^−4^	1.146 × 10^−3^	1.169 × 10^−3^	5.015 × 10^3^	1.912 × 10^4^	6.67 × 10^4^
S.D. of intercept (S_a_)	3.371 × 10^−4^	1.16 × 10^−3^	1.143 × 10^−3^	3.21 × 10^3^	1.377 × 10^4^	4.908 × 10^4^
S.D. of slope (S_b_)	6.828 × 10^−6^	1.794 × 10^−5^	9.583 × 10^−6^	1.667 × 10^2^	2.723 × 10^2^	7.00 × 10^2^
S.D.	0.94	1.51	1.28	0.44	1.49	1.10
% RSD^a^	0.95	1.53	1.29	0.44	1.49	1.10
% Error^b^	0.39	0.86	0.53	0.18	0.61	0.45
LOD (µg/mL)^c^	1.10	1.90	1.90	0.25	1.47	1.70
LOQ (µg/mL)^d^	3.40	5.80	5.50	0.76	4.40	5.25

^a^Percentage relative standard deviation

^b^Percentage relative error

^c^Limit of detection

^d^Limit of quantitation

## Results

### Third derivative spectrophotometric method

**Fig. 2:**
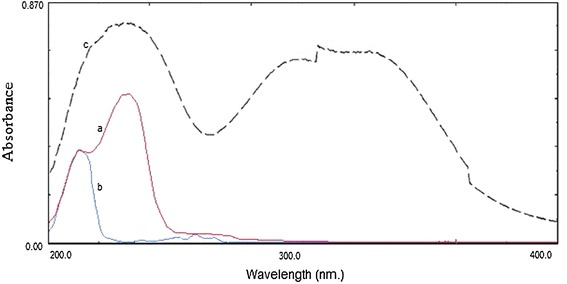
Absorption spectra of: (a) CTZ (b) PPA (c) NMS, conc. of each 20 µg/mL in methanol

**Fig. 3:**
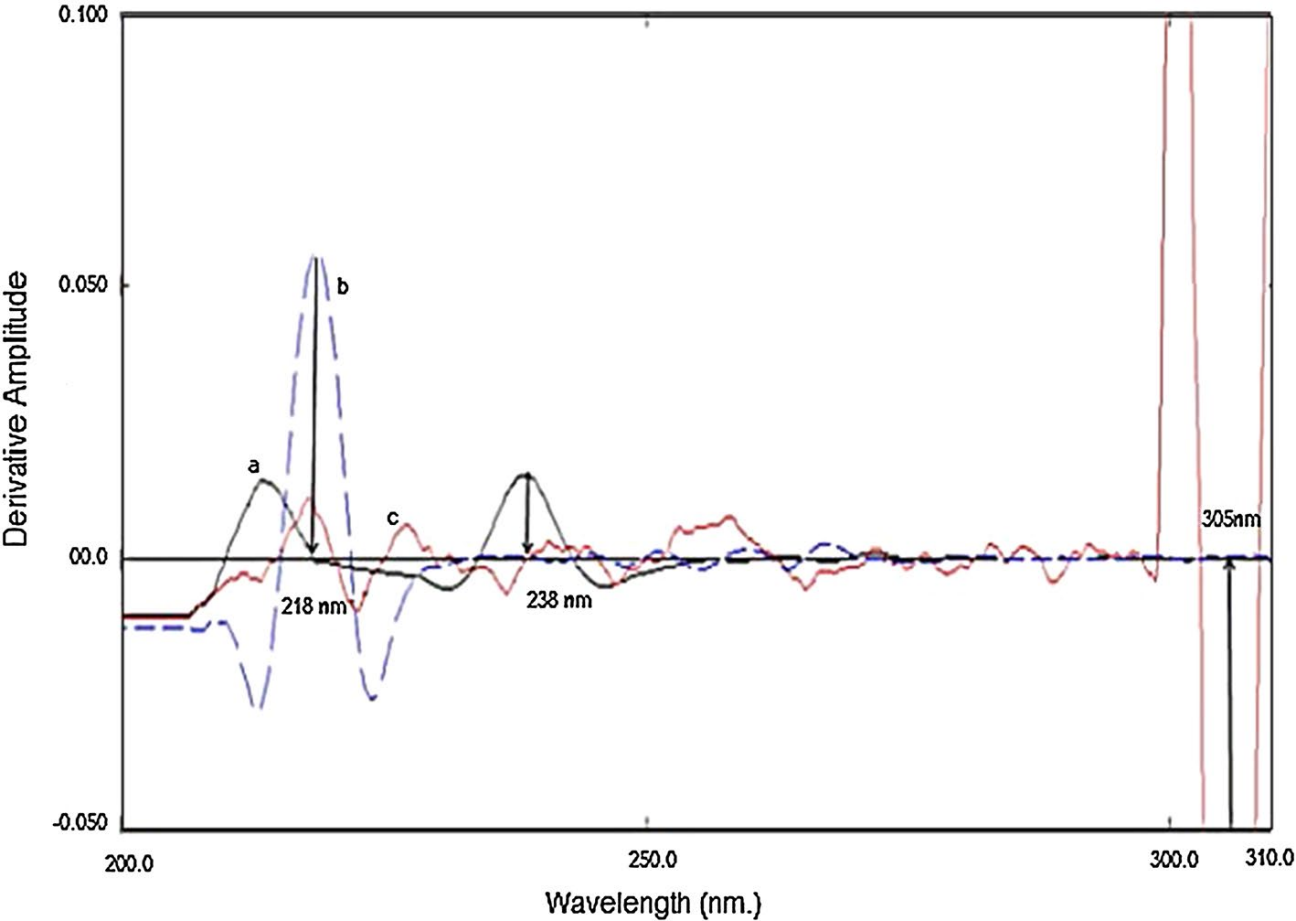
Third order derivative absorption spectra of: (a) CTZ (8 µg/mL), (b) PPA (40 µg/mL), (c) NMS (160 µg/mL) in methanol

**Fig. 4:**
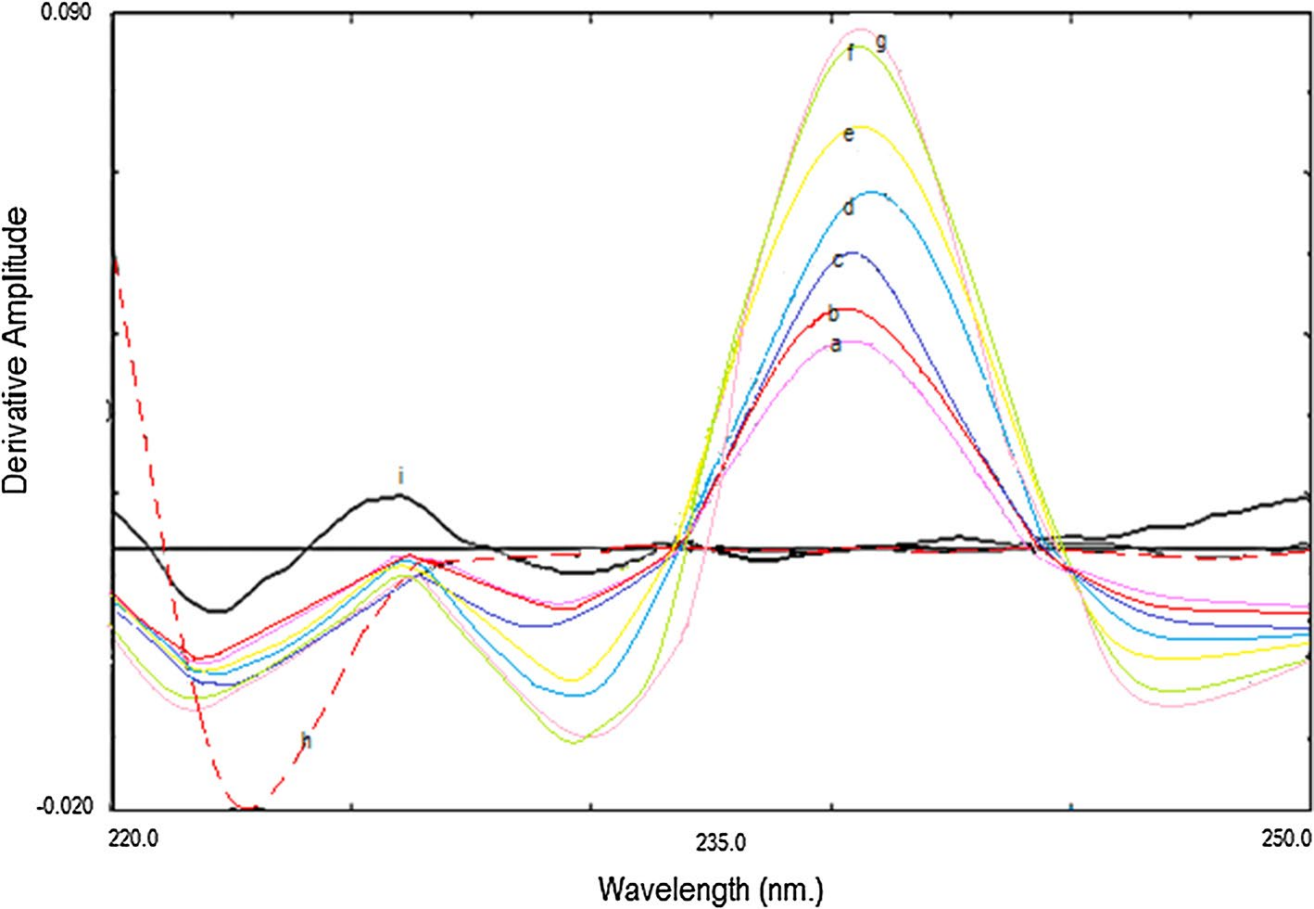
Third order derivative absorption spectra of: (a–g) CTZ (8, 10, 16, 20, 50, 60 and 90 µg/mL), (h) NMS (20 µg/mL), (i) PPA (20 µg/mL)

**Fig. 5:**
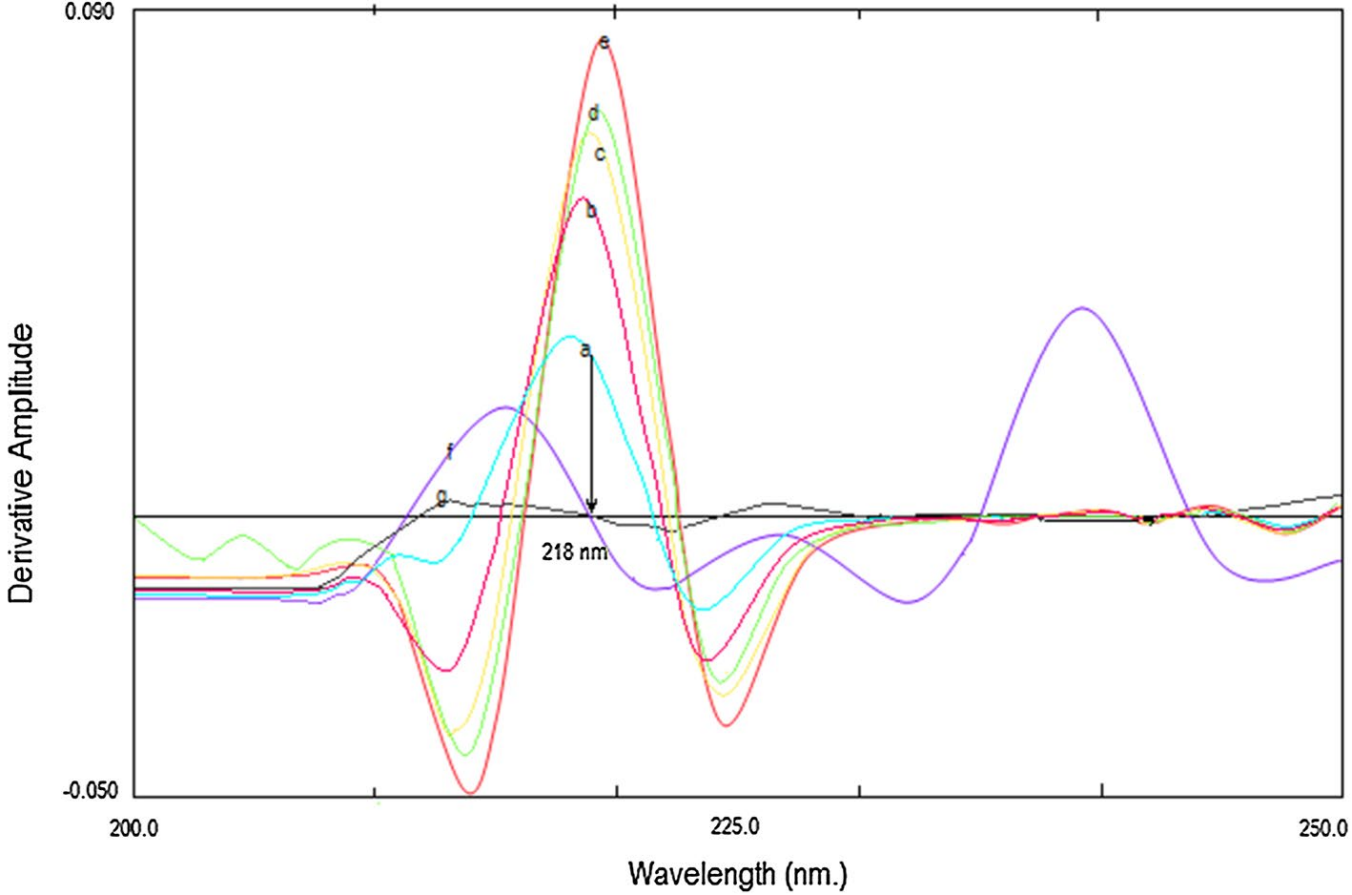
Third order derivative absorption spectra of: (a–e) PPA (20, 40, 50, 80 and 100 µg/mL), (f) CTZ (20 µg/mL), (g) NMS (20 µg/mL)

**Fig. 6:**
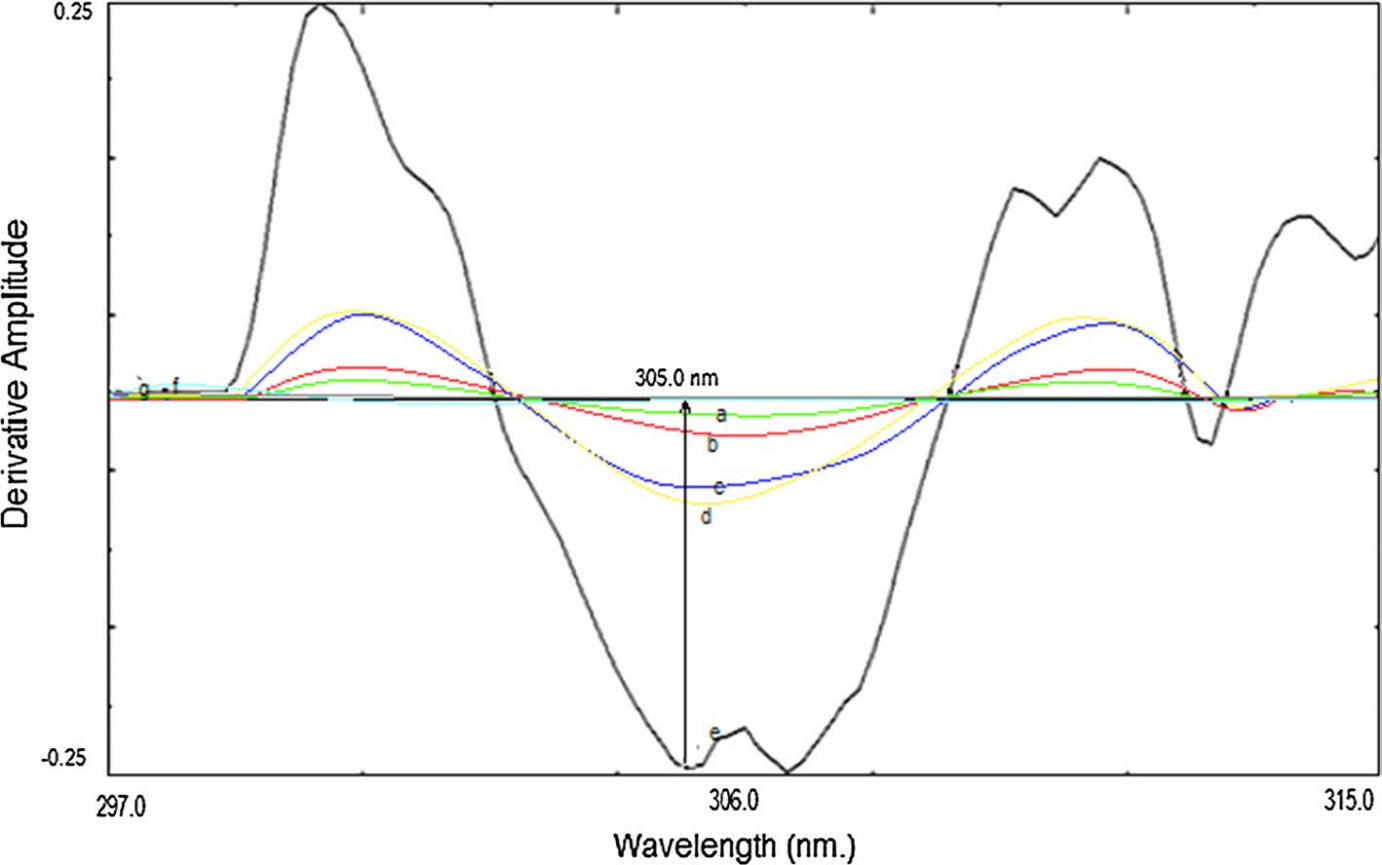
Third order derivative absorption spectra of: (a–e) NMS (20, 30, 40, 50 and 80 µg/mL), (f) CTZ (20 µg/mL), (g) PPA (20 µg/mL)

The simultaneous analysis of the three drugs by classical spectrophotometric method is a challenge owing to the strong overlapping of their zero order spectra (Fig. 2), and the difference between their concentrations in the tablet. Also there was strong overlapping in first and second order derivative spectra, third derivative spectrophotometry was used in the analysis of the three drugs mixture without interference from each other (Fig. 3). CTZ could be assayed by measuring its third derivative amplitude at zero crossing points of NMS and PPA at 238 nm (Fig. 4) and PPA could be determined at zero crossing points of CTZ and NMS at 218 nm (Fig. 5). Also NMS was determined at zero crossing points of CTZ and PPA at 305 nm (Fig. 6).

### Chromatographic method (HPLC)

#### Optimization of the chromatographic performance

**Fig. 7:**
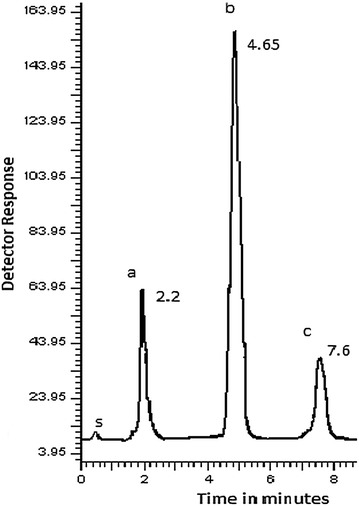
Typical chromatogram of laboratory prepared mixture under the described chromatographic conditions: (a) PPA (30 µg/mL), (b) NMS (120 µg/mL), (c) CTZ (6 µg/mL) (s) solvent front

**Fig. 8:**
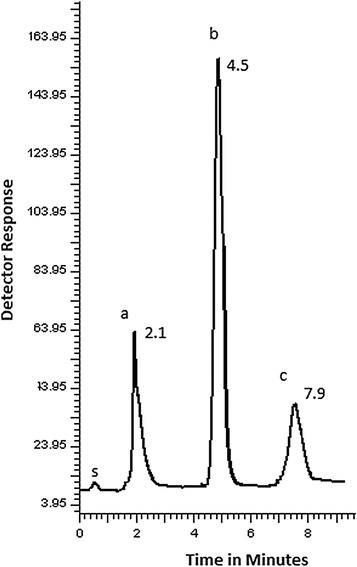
Typical chromatogram of laboratory prepared co-formulated tablet under the described chromatographic conditions: (a) PPA (30 µg/mL), (b) NMS (120 µg/mL), (c) CTZ (6 µg/mL) (s) solvent front

Studying of chromatographic conditions was carried out to reach the optimum conditions that achieve good and efficient separation. Figure 7 shows typical chromatogram for CTZ, PPA and NMS laboratory-prepared mixture and Fig. 8 shows the typical chromatogram for laboratory prepared tablet.

##### Column choice

Reversed-phase Chromolith^®^ performance (RP-18 monolithic, 100 mm × 4.6 mm i.d.) and Promosil ODS 100 A column (250 × 4.6 mm i.d. 5 µm particle size) were tried during the separation. The first column was the suitable one as it resulted in well resolved peaks in shorter time.

##### Appropriate wavelength choice

The UV absorption spectra of the studied drugs in methanol show maxima at 211 and 231 nm for CTZ, 218 nm for PPA and 238, 296 and 307 nm for NMS (Fig. 2). HPLC chromatograms for studied drugs were scanned from 200 to 400 nm to determine the suitable wavelength and it was found that 215 nm was the suitable wavelength as the studied drugs showed high absorbance at this wavelength especially CTZ as it found in low concentration in the tablet dosage form.

##### Mobile phase composition

Different modifications were done for the mobile phase to enhance the efficiency of the separation procedures as illustrated in Table 2.

**Table 2:** Optimization of the chromatographic conditions for separation of a mixture of cetirizine, phenylpropanolamine and nimesulide by the proposed HPLC method

Parameter	No. of theoretical plates (N)			Resolution (Rs)		Tailing factor (T)			Capacity factor (K’)			Selectivity factor (α)	
	CTZ	PPA	NMS	CTZ/NMS	NMS/PPA	CTZ	PPA	NMS	CTZ	PPA	NMS	CTZ/NMS	NMS/PPA
PH of the mobile phase													
3	1330	979	1947	1.2	3.8	1.26	1.39	1.25	2.1	0.33	1.5	1.4	4.5
4.6	1398	1246	1548	1.25	4.6	1.31	1.5	1.37	2.61	0.367	2.04	1.28	5.6
6	2351	1248	1490	2.1	4.68	1.30	1.35	1.30	3.47	0.74	2.56	1.36	3.45
*7*	*2432*	*1794*	*2804*	*3.8*	*5.1*	*1.19*	*1.11*	*1.23*	*4.5*	*1.05*	*2.8*	*1.64*	*2.6*
Conc. of phosphate buffer													
0.05	1947	1696	2497	1.1	4.1	1. 34	1.49	1.38	3.4	1.06	2.75	1.24	2.5
*0.1*	*2432*	*1794*	*2804*	*3.8*	*5.1*	*1. 19*	*1.11*	*1.23*	*4.5*	*1.05*	*2.8*	*1.64*	*2.6*
0.2	1146	1280	1855	1.9	3.66	1.23	1.52	1.36	3.4	0.58	2.05	1.7	3.55
Conc. of methanol (% v/v)													
50%	1513	1309	2133	2.1	4.1	2.07	0.99	1.32	4.78	1.1	3.4	1.4	3.3
*60%*	*2432*	*1794*	*2804*	*3.8*	*5.1*	*1.19*	*1.11*	*1.23*	*4.5*	*1.05*	*2.8*	*1.64*	*2.6*
70%	2396	1271	1496	0.5	3.8	2.22	1.9	1.32	2.47	1.02	2.3	1.07	2.25
80%	1638	1229	1369	0.74	2.1	1.23	1.09	1.33	1.86	0.99	1.62	1.15	1.64
Type of organic modifier													
*Methanol*	*2432*	*1794*	*2804*	*3.8*	*5.1*	*1.19*	*1.11*	*1.23*	*4.5*	*1.05*	*2.8*	*1.64*	*2.6*
Acetonitrile	2278	1374	1795	2.1	4.1	1.36	0.77	1.27	3.2	0.5	2.03	1.59	4.6
n-Propanol	1920	900	1058	2.4	3.9	3.22	1.9	2.3	2.88	0.42	1.88	1.5	4.5
Flow rate (mL/min)													
0.8	1889	1123	2543	2.4	3.9	1.2	1.56	1.28	3.4	0.98	1.8	1.88	1.84
*1.0*	*2432*	*1794*	*2804*	*3.8*	*5.1*	*1.19*	*1.11*	*1.23*	*4.5*	*1.05*	*2.8*	*1.64*	*2.6*
1.2	2117	1247	2178	1.1	2.9	1.32	1.56	1.35	2.9	1.00	2.3	1.3	2.30

Italic values indicate the optimum chromatographic conditions

Number of theoretical plates (N) = ${5.54} \left( {\tfrac{{\text{tR}}}{{\text{Wh/2}}}} \right)^{2}$

Resolution (R_s_) = $$\frac{{2\Delta {\text{t}}_{\text{R}} }}{{{\text{W}}_{1} + {\text{W}}_{2} }}$$

Tailing factor (T) = $$\frac{{{\text{W}}_{0.05} }}{2f}$$

Selectivity factor (relative retention) (α) = $\frac{{\text{tR2} - \text{tm}}}{{\text{tR1} - \text{tm}}}$

Capacity factor (K’) = $$\frac{{\text{tR} - \text{tm}}}{{\text{tm}}}$$

Type of organic modifier

Upon studying different organic solvents; it was found that acetonitrile and n-propanol showed overlapping between solvent peak and PPA giving split peak. Methanol was selected for optimum chromatographic conditions, as it gave higher number of theoretical plates with well resolved sharp peaks.

Ratio of organic modifier

The mobile phase which gives rapid separation of CTZ, PPA and NMS in good resolution is methanol: 0.1 M phosphate buffer in the ratio (60: 40, v/v). As the ratio of methanol increased the retention time of CTZ, PPA and NMS was decreased. The ratios 70 and 80% v/v of methanol caused overlapping between CTZ and NMS. CTZ band broadening was observed with ratio 50% (Table 2).

Ionic strength of phosphate buffer

0.1 M phosphate buffer was used as it gaves the highest number of theoretical plates with good resolution. Decreasing or increasing the ionic strength of phosphate buffer results in lower resolution or overlapping peaks.

### Validation of the method

#### Data analysis

A linear relationship was established by plotting either the peak area or the derivative amplitude against the drug concentration in µg/mL for the HPLC and the spectrophotometric method respectively. The ranges of linearity were shown in Table 1. Equations referred to linear regression analysis are explained here:

Third derivative spectrophotometric method:

$$\begin{aligned}^{ 3}{\text{D}}_{ 2 3 8}&= 0.00 6 2+ 0.00 1 {\text{ C}}\,\;\;\;\;\;\;\;\;\left({{\text{r }}= \, 0. 9 9 9 9} \right)\quad{\text{for CTZ}}\\^{ 3} {\text{D}}_{ 2 1 8} &= \, - 0.0 2 8 3+ 0.00 2 {\text{ C}}\;\;\;\;\;\;\left({{\text{r }}= \, 0. 9 9 9 9} \right)\quad{\text{for PPA}} \\^{ 3} {\text{D}}_{ 30 5}& = \, - \, 0.0 3 6 2 { } + \, 0.00 2 {\text{C}}\;\;\;\;\;\;\left({{\text{r }}= \, 0. 9 9 9 9} \right)\quad{\text{for NMS}}\\ \end{aligned}$$

where: (^3^D_wavelength_) is the third derivative amplitude of the spectra at the cited wavelength, and (C) is the concentration in µg/mL.

HPLC method:

$$\begin{aligned} {\text{P }}&= 1 30 2 4+ 4 2 3 9 9 \;{\text{C}}\quad\;\;\;\;\left({{\text{r }} = \, 0. 9 9 9 9} \right)\quad{\text{for CTZ}}\\ {\text{P }} &= 4 9 2 5 6 2. 9+ 3 10 1 5\;{\text{C}}\quad\left({{\text{r }} = \, 0. 9 9 9 8} \right)\quad{\text{for PPA}}\\ {\text{P }} &= - { 72167} + 9 3 4 2 8 \;{\text{C}}\quad\;\;\left({{\text{r }} = \, 0. 9 9 9 9} \right)\quad{\text{for NMS}} \\ \end{aligned}$$

where: P is the peak area, C is the concentration of the drug in µg/mL and r is the correlation coefficient.

Theoretical basis assumes that the standard curve may be close to the origin, but practically it is rather difficult due to the presence of a reading for the solvent or the blank reading. As the intercept decreases in the calculations, this reflects that the solvent reading is almost near to zero [21]. Linearity of the calibration curves was proved through statistical analysis [21] of the data (Table 1).

The limit of quantitation and limit of detection were calculated according to ICH recommendations [22].

$${\text{LOQ }} = { 1}0{\text{ S}}_{\text{a}} /{\text{b}}\quad\;\;\;\;\;\;\;{\text{LOD }} = { 3}. 3 {\text{ S}}_{\text{a}} /{\text{b}}$$

where S_a_ is the standard deviation of the intercept of the calibration curve and b is the slope of the calibration curve. LOQ and LOD values for CTZ, PPA and NMS by the proposed methods were mentioned in Table 1.

In terms of accuracy; the results generated from the proposed methods were compared with those of well-established previous reports methods. The comparison method for CTZ and PPA describes reversed phase HPLC method [6] for simultaneous determination of both drugs using C_18_ column with UV detection at 217 nm. Concerning comparison method for determination of NMS; HPLC method [15] was utilized acetonitrile: 0.05M KH_2_PO_4_. The detection was carried out at 230 nm on C_18_ column. Accuracy was assessed through comparing the results of the proposed and the comparison methods and there was non-significant difference between the performance of them (Table 3). The ratio of CTZ, PPA and NMS in the tablet is not covered in the comparison method.

**Table 3:** Assay results for the determination of the studied drugs in pure form by the proposed and comparison methods

Compound	3rd derivative method			HPLC method			Comparison methods [6, 15]		
	Amount taken (μg/mL)	Amount found (μg/mL)	% Found	Amount taken (μg/mL)	Amount found (μg/mL)	% Found	Amount taken (μg/mL)	Amount found (μg/mL)	% Found
CTZ	8.00	7.9	98.75	5.00	4.905	98.10	5.00	4.98	99.58
	10.00	10.00	100.00	6.00	5.918	98.63	7.00	7.04	100.59
	16.00	16.9	99.38	8.00	8.036	100.45	9.00	8.98	99.77
	50.00	49.0	98.00	10.00	10.051	100.51			
	60.00	59.9	99.83	20.00	20.172	100.86			
	90.00	88.00	97.78	40.00	39.918	99.80			
Mean			98.96			99.73			99.98
± S.D.			0.94			0.44			0.58
*t*			1.72			0.365			
*F*			3.04			4.36			
PPA	20.00	20.00	100.0	10.00	9.842	98.42	10.00	9.898	98.98
	30.00	29.57	98.58	25.00	24.932	99.73	11.00	11.204	101.85
	40.00	39.5	98.75	30.00	30.334	101.11	12.00	11.898	99.15
	50.00	48.5	97.00	35.00	34.263	97.89			
	80.00	78.5	98.13	50.00	50.877	101.75			
	100.00	99.0	99.0	100.0	99.8	99.75			
Mean			98.58			99.78			99.99
± S.D.			1.23			1.49			1.61
*t*			1.66			0.203			
*F*			2.60			1.17			
NMS	20.00	20.0	100.0	10.00	10.10	101.07	50.00	50.71	101.42
	30.00	29.50	98.33	30.00	30.257	100.86	70.00	68.82	98.31
	40.00	39.00	97.50	40.00	40.254	100.64	100.00	100.47	100.47
	100.00	100.5	100.5	50.00	49.482	98.96			
	180.00	179.50	99.72	100.00	99.048	99.05			
	200.00	198.00	99.00	120.00	120.85	100.71			
Mean			99.18			100.22			100.07
± S.D.			1.28			1.1			1.53
*t*			0.989			0.179			
*F*			2.02			2.8			

Each result is the average of three separate determinations

The value of tabulated *t* and *F* are 2.20 and 19.29, respectively at P = 0.05 [21]

Repeatability and intermediate precision were tested to verify the precision of the proposed methods and the results were summarized in Table 4.

**Table 4:** Precision data for the determination of the studied drugs by the proposed methods

Parameters	Intra-day			Inter-day		
	$\overline{\text{x}} \pm {\text{S}} . {\text{D}}$	% RSD	% Error	$\overline{\text{x}} \pm {\text{S}} . {\text{D}}$	% RSD	% Error
3rd Derivative method						
CTZ (μg/mL)						
8	99.04 ± 1.04	1.05	0.61	100.05 ± 0.24	0.24	0.14
20	98.04 ± 0.45	0.46	0.27	98.8 ± 0.27	0.27	0.16
40	97.65 ± 0.53	0.54	0.31	98.93 ± 0.25	0.25	0.15
PPA (μg/mL)						
20	98.89 ± 1.27	1.29	0.74	99.08 ± 0.85	0.86	0.49
50	100.7 ± 1.85	1.84	1.06	99.99 ± 1.42	1.42	0.82
100	99.2 ± 1.83	1.85	1.07	100.59 ± 1.18	1.17	0.68
NMS (μg/mL)						
40	98.23 ± 0.77	0.79	0.45	99.27 ± 1.09	1.1	0.63
100	99.27 ± 1.22	1.32	0.71	100.6 ± 0.6	0.60	0.34
120	98.32 ± 0.62	0.63	0.36	99.91 ± 1.02	1.02	0.59
HPLC method						
CTZ (μg/mL)						
8	98.63 ± 0.95	0.96	0.56	100.53 ± 0.68	0.67	0.39
20	98.87 ± 0.49	0.50	0.29	100.75 ± 0.4	0.39	0.23
40	98.18 ± 0.47	0.48	0.27	98.15 ± 1.1	1.12	0.65
PPA (μg/mL)						
20	98.23 ± 0.55	0.56	0.32	99.53 ± 0.49	0.5	0.29
50	98.07 ± 0.15	0.16	0.09	99.88 ± 0.17	0.17	0.10
100	98.23 ± 0.83	0.83	0.49	98.94 ± 0.21	0.22	0.12
NMS (μg/mL)						
40	98.52 ± 0.62	0.63	0.36	98.77 ± 0.42	0.42	0.24
100	98.67 ± 0.36	0.36	0.21	99.45 ± 0.52	0.52	0.30
120	98.52 ± 0.95	0.96	0.55	100.24 ± 0.87	0.87	0.5

Each result is the average of three separate determinations

#### Robustness (for the HPLC method)

Some variables were changed on constancy of others for robustness investigation. These variables included; pH (6.9 ± 0.1) and phosphate buffer concentration (0.1 ± 0.005 M). These small changes had no effect on the separation and resolution of CTZ, PPA and NMS. This gave a good indication for the reliability of the proposed method.

### Application in pharmaceutical preparations

#### Analysis of laboratory prepared mixtures

A successful determination for the three drugs in their laboratory prepared mixtures was performed and summarized in Table 5.

**Table 5:** Assay results for the determination of the studied drugs in different synthetic mixtures in different pharmaceutical ratios

Parameter	Amount taken (μg/mL)			Proposed method						Comparison methods [6, 15]					
				Amount found (μg/mL)			% Found			Amount taken (μg/mL)			% Found		
	CTZ	PPA	NMS	CTZ	PPA	NMS	CTZ	PPA	NMS	CTZ	PPA	NMS	CTZ	PPA	NMS
3rd Derivative method	8.0	40.0	160.0	7.8	40.0	157.0	97.5	100.0	98.13	5.00	10.0	15.0	99.18	99.77	101.3
	9.0	45.0	180.0	8.8	45.0	180.5	98.89	100.0	100.3	5.50	11.0	16.5	100.3	100.9	100.5
	10.0	50.0	200.0	9.7	49.0	199.0	98.0	98.00	99.50	6.00	12.0	18.0	101.8	99.43	98.99
	12.0	24.0	36.0	12.2	23.5	40.0	101.7	97.92	100.0	8.00	8.00	8.00	98.09	99.81	99.49
	40.0	40.0	40.0	39.1	40.0	40.0	100.0	100.0	100.0	10.0	10.0	10.0	100.7	99.77	99.63
Mean							99.21	98.18	99.59				100.0	99.94	99.98
± S.D.							1.67	1.18	0.95				1.54	0.80	0.34
*t*							1.22	0.139	0.70						
*F*							1.39	1.16	1.12						
HPLC method	5.0	25.0	100.0	4.89	39.95	98.44	97.70	100.2	98.44	5.00	10.0	15.0	99.18	99.77	101.3
	5.5	27.5	110.0	5.57	44.11	110.2	101.3	100.6	100.2	5.50	11.0	16.5	100.3	100.9	100.5
	6.0	30.0	120.0	6.02	50.60	121.05	100.4	99.45	100.9	6.00	12.0	18.0	101.8	99.43	98.99
	12.0	24.0	36.00	12.0	23.7	36.64	100.3	99.64	101.8	8.00	8.00	8.00	98.09	99.81	99.49
	40.0	40.0	40.00	39.9	39.5	39.69	99.98	99.98	99.25	10.0	10.0	10.0	100.7	99.77	99.63
Mean							99.91	99.97	100.1				100.0	99.94	99.98
± S.D.							0.55	0.53	1.06				1.57	0.80	0.34
*t*							0.14	0.122	0.24						
*F*							1.16	1.51	2.1						

Each result is the average of three separate determinations

The value of tabulated *t* and *F* are 2.13 and 6.4 respectively at P = 0.05

#### Dosage form analysis

Co-formulated tablets was also analyzed using the proposed HPLC and spectrophotometric methods as illustrated in Table 6. The results of statistical analysis were satisfactory as indicated by Student’s *t* test and variance ratio *F* test [21].

**Table 6:** Assay results for the determination of the studied drugs in their laboratory prepared co-formulated tablets

Parameter	Amount taken (μg/mL)			Proposed method						Comparison methods [6, 15]					
				Amount found (μg/mL)			% Found			Amount taken (μg/mL)			%Found		
	CTZ	PPA	NMS	CTZ	PPA	NMS	CTZ	PPA	NMS	CTZ	PPA	NMS	CTZ	PPA	NMS
3rd Derivative method	8.0	40.0	160.0	7.9	40.0	161.5	98.75	100.0	98.13	5.0	10.0	90.0	99.72	99.29	98.08
	9.0	45.0	180.0	9.01	45.5	183.0	100.1	101.1	99.44	6.0	11.0	95.0	100.5	101.3	97.26
	10.0	50.0	200.0	9.9	50.1	202.0	99.00	100.2	98.2	7.0	12.0	100.0	99.80	99.41	99.5
Mean							99.29	100.4	98.6				100.0	99.99	98.28
± S.D.							0.72	0.14	0.74				0.46	1.12	0.46
*t*							1.48	1.17	0.397						
*F*							2.85	1.05	2.36						
HPLC method	5.0	25.0	100.0	4.97	25.10	101.05	99.32	100.4	101.1	5.0	10.0	90.0	99.72	99.29	98.08
	5.5	27.5	110.0	5.57	27.3	121.05	101.2	99.3	100.9	6.0	11.0	95.0	100.5	101.3	97.26
	6.0	30.0	120.0	5.97	30.1	107.9	99.43	100.2	98.09	7.0	12.0	100.0	99.8	99.41	99.5
Mean							99.98	99.97	100.0				100.0	99.99	98.28
± S.D.							1.27	0.61	1.66				0.46	1.12	0.46
*t*							0.035	0.045	1.49						
*F*							6.046	3.7	2.205						

Each result is the average of three separate determinations

The value of tabulated *t* and *F* are 2.92 and 19.00 respectively at P = 0.05 [21]

## Discussion

Third derivative spectrophotometry was used to analyze CTZ, PPA and NMS without interference from each other (Fig. 3). This method is simple, sensitive and efficient alternative to spectrophotometric methods mentioned for each of the three drugs in the literature [9, 13, 18], as it doesn’t need any reagents or additional time consuming steps.

The proposed approach also describes a novel HPLC method for the simultaneous determination of CTZ, PPA and NMS on a monolithic column. The established method is capable to separate the drugs with high efficacy and high resolution factor and within a short analysis time.

## Conclusion

The current work provides the first method for the simultaneous analysis of CTZ, PPA and NMS in their pharmaceutical formulations. The developed spectrophotometric method is simple, rapid and economic. The HPLC method is a sensitive, reliable and time-saving method where separation of the studied analytes is achieved in less than 8 min. Moreover, the proposed methods overcome the analytical problems raised by the ratio of CTZ, PPA relative to NMS (1:5:20) and therefore could be used in the analysis of their co-formulated tablets in quality control laboratories.

## Abbreviations

CET: cetirizine
PPA: phenylpropanolamine
NMS: nimesulide
ICH: international conference on harmonization
LOQ: limit of quantification
LOD: limit of detection
